# Maternal health care service utilization in post-war Liberia: analysis of nationally representative cross-sectional household surveys

**DOI:** 10.1186/s12889-018-6365-x

**Published:** 2019-01-08

**Authors:** Sanni Yaya, Olalekan A. Uthman, Ghose Bishwajit, Michael Ekholuenetale

**Affiliations:** 1 School of International Development and Global Studies, University of Ottawa, Ottawa, Canada; 2 Warwick Centre for Applied Health Research and Delivery (WCAHRD), Division of Health Sciences, Warwick Medical School, University of Warwick, Coventry, CV4 7AL UK; 3 Department of Epidemiology and Medical Statistics, Faculty of Public Health, College of Medicine, University of Ibadan, Ibadan, Nigeria

**Keywords:** Liberia, Post-war era, Maternal healthcare service utilization, Antenatal care, Facility-based delivery

## Abstract

**Background:**

Post-war Liberia has a fast-growing population and an alarming maternal mortality ratio (MMR). To provide a better understanding about healthcare system recovery in post-war country, we explored the changes in maternal healthcare services utilization between 2007 and 2016.

**Methods:**

We used 2007 and 2013 Liberia Demographic and Health Survey (LDHS) and the 2016 Malaria Indicator Survey (MIS) in this study. The outcomes of interest were: place of delivery and antenatal care visits. Univariate analysis was conducted using percentages and means (standard deviations) and multiple binary multivariable logistic models were used to examine the factors associated with the outcome variables.

**Results:**

Between 2007 and 2016, the percentage of adequate ANC visits increased from 71.20 to 79.8%, and that of facility-based delivery increased from 40.90 to 74.60%. The odds of attending at least four ANC visits and formal institutional delivery were low among women residing in rural area, but high among women with higher education, used electronic media, and lived in high wealth index households. Additionally, attending ANC at least four times increased the odds of facility-based delivery by almost threefold.

**Conclusion:**

The findings suggest that key maternal healthcare utilization indicators have improved substantially, especially facility-based delivery. However, a large proportion of women remain deprived of these life-saving health services in the post-war era. Greater healthcare efforts are needed to improve the quality and coverage of maternal healthcare in order to enhance maternal survival in Liberia.

## Background

Globally, over 90% of maternal and child mortalities occur in developing countries, with the most vulnerable populations sharing the greatest burden of the mortality from preventable causes [1, 2]. No or inadequate access to maternal healthcare services increases the risks of a range of pregnancy and obstetric complications that can result in higher rates of maternal morbidity and mortality that in turn lowers the chances of child survival. In Africa, health care system has not satisfactorily met the healthcare needs of women. Maternal mortality in sub-Saharan region has remained unacceptably high, recording over 510 in maternal mortality cases per 100,000 live births [3]. For decades, there has been increasing interest in the provision and accessibility to maternal health care services in developing countries, particularly in those emerging from long years of civil war, such as Liberia. The interest is to make concerted efforts in reducing maternal and neonatal mortality, which has recorded persistent high prevalence [1].

Liberia ended 14 years of civil war in 2003. In the course of the war, the health care system was devastated where preponderance health facilities were rendered ineffective, which has made the country to account for some of the poorest health outcomes. This large breakdown in the health system brought increased dependence on alternative health care services, such as traditional healers amongst others [4]. A remarkable event of the two consecutive Liberian civil wars (between 1989 and 1996 and 1997–2003 respectively) is the deterioration of the country’s health system care system [5]. Liberia is currently identified among the countries in the world with the highest maternal mortality ratios, with an alarming rate of over 725 deaths per 100,000 live births [6]. Although the neonatal, infant and under-five mortality have reportedly declined over the past decade, the rates are still very high for infants (54 deaths per 1000 live births) and under-five children (approximately 1 in 11 children) [7]. These indicators place Liberia among the sub-Saharan African countries with highest maternal and neonatal mortality rates. Like other post-war countries, health care system in Liberia faces a multitude of supply side barriers such as inadequate infrastructure, lack of skilled health personnel, and medical equipment [8]. Low utilization of contraceptive methods and high teenage pregnancies are prominent contributors to maternal death, as approximately 60% of neonatal deaths occur among adolescent women compared to about 6% for those above 19 years, which indicates the need to delay child birth through access to improved sexual and reproductive health care services [9]. The health sector has made continuous efforts to enhance overall access to health services in Liberia. In 2007, Liberian government initiated a widespread effort to prioritize access to basic health care and rebuild the health care system [10]. Basic Package of Health Services (BPHS) was constructed as cost-effective and evidence-based interventions, necessary to improve maternal health by identifying high-priority services in health care facilities [11]. This approach was adopted in other post-war countries, such as Afghanistan, Sierra Leone and South Sudan, and has been recognized with increasing access to health care and reduction morbidity and mortality [12–15]. A previous study showed that, less than 50% of all deliveries in Liberia were conducted by a skilled birth attendant, with a large disparity by geographical locations. The least skilled birth attendance was in the northern central region of the country [16].

Currently, there are serious demand side constraints, such as misconceptions or wrong perception, lack of awareness or knowledge and harmful socio-cultural norms in the provision of and accessing maternal healthcare services especially for the socioeconomically disadvantaged populations. The National Drug Service (NDS) is yet to meet required operational standard, notwithstanding an overall overhaul is in progress especially in delivery of necessary RMNCAH services. This approach is an essential component of Liberia’s plans and policies to achieve the Sustainable Development Goals (SDGs), particularly goals 3 and 5 by year 2030 [17]. It also states the country’s efforts in line with United Nation’s scheme of Every Woman, Every Child initiative to drastically reduce maternal and child mortality from preventable causes, as contained in the regulatory framework [9, 18].

The RMNCAH framework centers on sustainable health outcomes, based on the principles of promoting equity and gender equality, ownership of the health system at national levels, respect for reproductive health rights, equality in access to health care utilization and ensuring a responsive health system to patients’ needs. The major approach to optimize efficiency in health care delivery is linked to enhanced accountability through improved productivity, performance incentives and integrating RMNCAH service delivery with other health care programs, such as malaria, tuberculosis, HIV amongst others [18]. In this paper, our goal is to explore the determinants of changes in maternal services utilization by deprived groups in post-conflict settings.

## Methods

### Data source

Datasets used in this study were obtained from the 2007 and 2013 rounds of Liberia Demographic and Health Surveys (LDHS) and 2016 Malaria Indicator Survey (MIS). Questionnaire for individual women aged 15–49 was used to obtain nationally representative sample respondents. The population-based surveys were commissioned by USAID and carried out by the Liberia government, with operational support from ICF International. The surveys employ stratified two-stage cluster design. Clusters or Enumeration Areas (EA) were first selected from recent population census sample frame and households were systematically selected from the clusters. The surveys used standardized questionnaires developed by the MEASURE DHS programme particularly for households, men and women these are administered during face-to-face interviews. The response rates were above 95% for each survey; as such it was unnecessary to conduct weighting prior to data analysis. Details of survey procedures can be found elsewhere [6, 19].

### Study sample

The data for this study was extracted from the Liberia population-based surveys for the years 2007, 2013 and 2016. The individual women datasets were used in the analysis; these consisted of large representative samples of women aged 15–49 years. Data from 7092 women in 2007, 9239 women in 2013, 4290 women in 2016 were analyzed in this study.

### Variables measurement and conceptual framework

The main outcome variables are ANC visits and place of delivery. The number of ANC visits is the self-reported frequency of skilled care attention or treatment received during pregnancy. Also, the place of delivery is used to identify where childbirth occurred, this was in dichotomous forms; whether at respondents’ home/other traditional homes or at a health facility. In addition, two groups of independent variables were used, namely; background and maternal characteristics. The selection of explanatory variables was based on the conceptual framework by Ejembi et al. [20]. The contextual factors implicitly influence maternal sexual and reproductive health care by acting on the individual and household factors at various levels to influence women’s health care service utilization. The background characteristics included maternal age (measured in the interval; 15–19, 20–24, 25–29, 30–34, 35–39, 40–44 and 45–49), region, place of residence (whether urban or rural), education (including women with no formal education, up to those with higher education), religion, sex of household head and wealth index. The wealth index was constructed using household asset data, such as ownership of items including; source of drinking water, sanitation facilities, television, bicycle, car and type of flooring material. DHS used information on household assets to create an index that is used to represent the wealth of the households [6, 20, 21]. The proximate characteristics were age at first birth, children ever born and maternal frequency of reading newspaper or magazine, listening to radio and watching television.

### Data analysis

In this paper, we used univariate analysis to summarize respondents’ characteristics from the three rounds of the data sets using percentages and mean (and standard deviations). To adjust for complex survey design, we used the survey module (svyset) for all analyses to account for clustering, stratification and sample weight. Correlation matrix was used to conduct multicollinearity diagnostics to identify interdependence between explanatory variables using a cut-off of 0.6 [22]. Hence, all explanatory variables were retained for analysis due to lack of collinearity. In addition, Mckelvey and Zavoinas model fitness was presented [23]. the trend of maternal health care in post-war era was presented in chart. We fitted the unadjusted and adjusted binary logistic regression models to assess significant factors of facility-based delivery and antenatal care visits. Measures above 1 signified a higher likelihood of maternal healthcare use, and measures less than 1 means a lower likelihood of maternal healthcare use. Statistical significance was set at 5%, and analyses were conducted using Stata version 14.0 (Statacorp, College Station, Texas, United States of America).

### Ethical considerations

We did the analyses using publicly available data from demographic health surveys. Ethical procedures were the responsibility of the institutions that commissioned, funded, or managed the surveys. All DHS surveys are approved by ICF international as well as an Institutional Review Board (IRB) in respective country to ensure that the protocols follow the U.S. Department of Health and Human Services regulations for the protection of human subjects.

## Results

### Study participant characteristics

Results presented in Table 1 showed the summary statistics of the characteristics of respondents from 2007, 2013 and 2016 surveys. The mean age (SD) of respondents was approximately 29 (9.0) years. Also, the mean age (SD) at first birth was 18(3.0) years for the surveys. For age interval, respondents aged 40–44 and 45–49 years were least represented below one-tenth each. While participants ages; 15–19, 20–24 and 25–29 years were almost equally represented about 20% each. In 2007, majority of respondents were from Morovia, followed by North Central region, but in 2013, about 30% of the respondents were from South Central and followed by North central. In 2016, vast respondents were majorly from Morovia. This shows that Morovia is largely populated which proportionately led to more selection of respondents compared to other regions. Furthermore, the highest proportion of respondents from urban place of residence was 54.3%. This indicates that about half of respondents are rural dwellers. About 40% of participants have no formal education; up to 30% have only primary education. The trend (2007–2016) showed that approximately one-third have at least secondary education. On religious beliefs, about 88% are Christians, and 10% are Muslims. The economic status of the respondents was measured using the wealth index, this revealed that a maximum of 52% are below middle class, while less than half was ever above middle class. See Table 1 for details.

**Table 1:** Characteristics of respondents, Liberia, 2007–2016

Variable, %(95% CI)	2007 DHS (*n* = 7092)	2013 DHS (*n* = 9239)	2016 MIS (*n* = 4290)
Mean age (SD)	29.2 (9.6)	29.2 (9.8)	28.7 (9.4)
Mean age at first birth (SD)	18.6 (3.7)	18.2 (3.4)	No data
Age			
15–19	18.9 (18.0–19.8)	20.7 (19.9–21.6)	20.9 (19.7–22.1)
20–24	19.5 (18.6–20.5)	17.1 (16.4–17.9)	18.6 (17.5–19.8)
25–29	16.4 (15.5–17.3)	17.2 (16.4–17.9)	15.8 (14.8–17.0)
30–34	14.1 (13.4–15.0)	13.5 (12.8–14.2)	15.8 (14.7–17.0)
35–39	13.1 (12.3–13.9)	13.0 (12.3–13.7)	12.5 (11.6–13.5)
40–44	9.5 (8.8–10.2)	9.8 (9.2–10.4)	9.4 (8.6–10.4)
45–49	8.5 (7.9–9.2)	8.7 (8.2–9.3)	6.9 (6.2–7.7)
Region			
Morovia	26.2 (25.2–27.2)	–	21.3 (20.1–22.5)
North Western	10.8 (10.1–11.5)	16.9 (16.1–17.6)	12.2 (11.2–13.2)
South Central	15.1 (14.3–16.0)	29.9 (28.9–30.8)	17.0 (15.9–18.1)
South Eastern A	11.3 (10.6–12.1)	14.8 (14.1–15.5)	14.9 (13.9–16.0)
South Eastern B	17.5 (16.7–18.4)	15.5 (14.8–16.2)	17.4 (16.3–18.5)
North Central	19.0 (18.2–20.0)	23.0 (22.2–23.9)	17.3 (16.2–18.5)
Place of residence			
Urban	45.0 (43.9–46.2)	40.3 (39.3–41.3)	54.3 (52.8–55.8)
Rural	55.0 (53.8–56.1)	59.7 (58.7–60.7)	45.7 (44.2–47.2)
Level of education			
No education	41.8 (40.6–42.9)	39.8 (38.8–40.8)	35.5 (34.1–36.9)
Primary	35.0 (33.9–36.1)	34.6 (33.6–35.6)	27.6 (26.3–29.0)
Secondary	21.6 (20.6–22.6)	23.4 (22.6–24.3)	32.8 (31.4–34.2)
Higher	1.7 (1.4–2.0)	2.2 (1.9–2.5)	4.1 (3.5–4.7)
Religion			
Christian	87.1 (86.3–87.9)	85.2 (84.4–85.9)	87.9 (86.9–88.8)
Muslim	9.9 (9.2–10.6)	11.9 (11.2–12.6)	10.4 (9.5–11.4)
Traditional	0.4 (0.3–0.6)	0.4 (0.3–0.5)	0.4 (0.2–0.6)
Nil	2.6 (2.3–3.0)	0.3 (0.2–0.4)	1.3 (1.0–1.7)
Sex of household head			
Male	64.8 (63.7–65.9)	63.8 (62.8–64.7)	66.2 (64.7–67.6)
Female	35.2 (34.1–36.3)	36.2 (35.3–37.2)	33.8 (32.4–35.3)
Frequency of reading newspaper or magazine			No data
Not at all	77.2 (76.2–78.2)	84.6 (83.9–85.4)	
Less than once a week	7.9 (7.2–8.5)	9.4 (8.8–10.0)	
At least once a week	10.7 (10.0–11.4)	5.9 (5.5—6.4)	
Almost every day	4.3 (3.8–4.8)	–	
Frequency of listening to radio			No data
Not at all	40.0 (38.8–41.1)	33.5 (32.6–34.5)	
Less than once a week	11.2 (10.4–11.9)	31.4 (30.5–32.4)	
At least once a week	22.2 (21.3–23.2)	35.1 (34.1–36.1)	
Almost every day	26.7 (25.6–27.7)	–	
Frequency of watching television			No data
Not at all	61.6 (60.4–62.7)	73.5 (72.6–74.4)	
Less than once a week	10.2 (9.5–10.9)	14.9 (14.2–15.7)	
At least once a week	17.1 (16.2–18.0)	11.5 (10.9–12.2)	
Almost every day	11.2 (10.4–11.9)	–	
Wealth index			
Poorest	19.0 (18.1–19.9)	28.0 (27.1–28.9)	23.5 (22.2–24.8)
Poorer	19.5 (18.6–20.4)	24.7 (23.8–25.6)	18.7 (17.6–19.9)
Middle	19.7 (18.8–20.6)	21.6 (20.8–22.5)	22.6 (21.4–23.9)
Richer	22.1 (21.1–23.0)	14.1 (13.4–14.9)	18.3 (17.1–19.4)
Richest	19.8 (18.9–20.7)	11.6 (10.9–12.2)	16.9 (15.9–18.1)
Children ever born			
1–4	65.6 (64.4–66.8)	62.0 (60.9–63.1)	62.1 (60.5–63.8)
> 4	34.4 (33.2–35.6)	38.0 (36.9–39.1)	37.9 (36.2–39.5)

### Changes in maternal services utilization

**Fig. 1:**
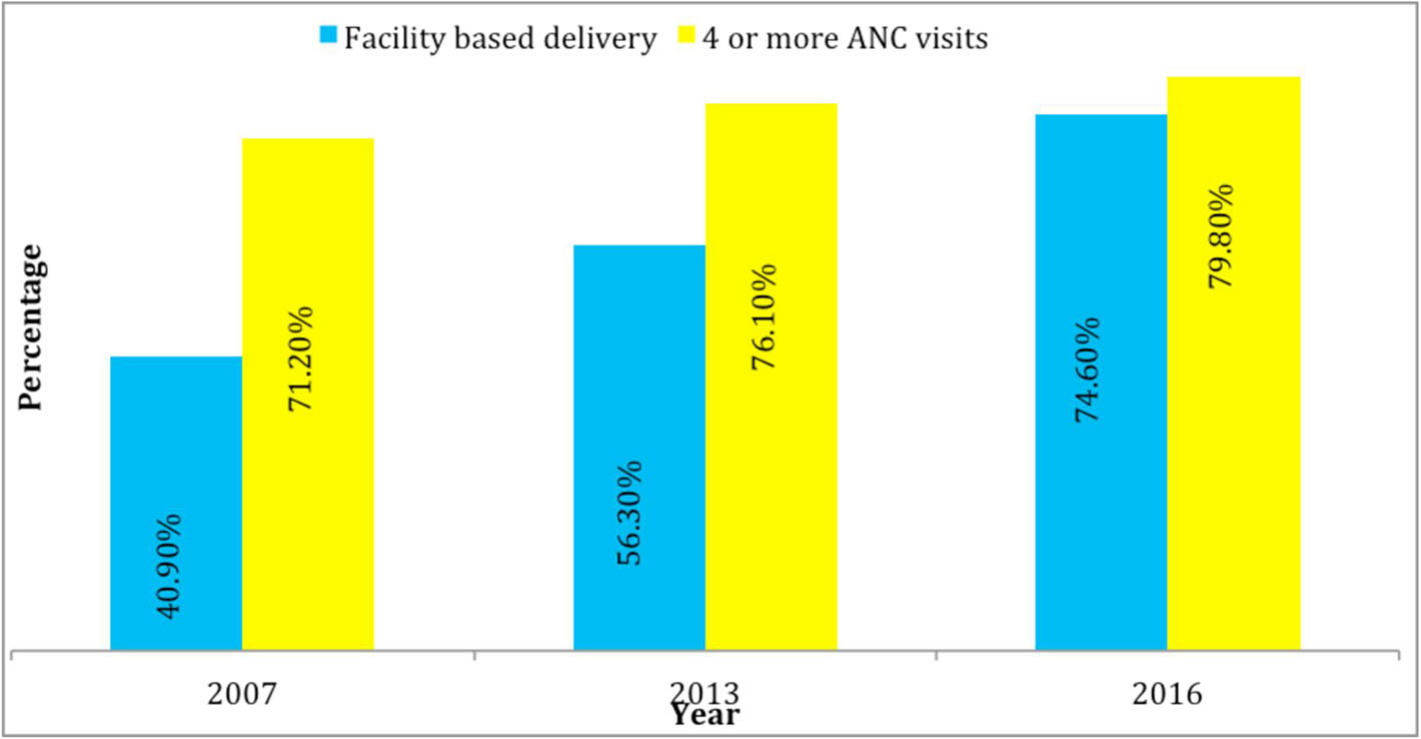
Changes in maternal services utilization 2007–2016

The trend of facility-based delivery showed a maximum of three-quarters in the post-war era. Figure 1 indicated 33% increase in a decade. However, ANC visits at least 4 showed a weak change in a decade, starting from 71.2% in 2007, to 76.1% in 2013 and now to 79.8% in 2016. Only 8.6% increase in a decade. The trend in facility-based delivery seems to have improved better compared to adequate ANC visits among respondents.

### Factors associated with facility-based delivery

The factors associated with facility-based delivery were examined using binary logistic regression in Table 2. There was significant increase in the odds of facility-based delivery in 2013 compared to 2007 (OR = 2.21; 95%CI = 1.30–3.75). Women aged 20–24 and 25–29 years had significant reduction in the odds of facility-based delivery compared to younger women aged 15–19 years. Regional diversity was associated with facility-based delivery, after adjusting for other covariates. In addition, rural women had 55% reduction in the odds of facility-based delivery when compared to their urban counterpart (OR = 0.45; 95% CI = 0.31–0.65). Maternal education improved the utilization of facility-based delivery as educated women were found to have higher odds when compared to women with no formal education. More so, women with no religious affiliation had reduction in facility-based delivery, compared to Christian women, after adjusting for other confounders. Households with female head had higher odds of facility-based delivery after controlling for other covariates (OR = 1.20; 95% CI = 1.07–1.36). The use of media specifically women who listen to radio significantly had increase in the odds of facility-based delivery compared to women who do not listen to radio; women who listen to radio almost every day were 1.61 times as likely to utilize facility-based delivery compared to non-listeners (OR = 1.61; 95% CI = 1.38–2.02); for those who watch television almost every day (OR = 1.41; 95% CI = 1.24–2.10). Women with middle, richer and richest wealth index had significant increase in the odds of facility-based delivery when compared to poorest women, after adjusting for other covariates. Antenatal care (ANC) visits was significantly associated with facility-based delivery; women who reported at least 4 ANC visits were 2.90 times as likely to utilize facility-based delivery, compared to women who reported less than ANC visits (OR = 2.90; 95%CI = 2.52–3.36). Further results showed the interaction between year and place of residence; respondents from year 2013 and rural residence were 1.64 times as likely to utilize facility-based delivery compared to respondents from 2007 and in urban residence. See Table 2 for details.

**Table 2:** Multivariable logistic regression model to examine factors associated with facility-based delivery in Liberia, 2007–2016

Variable	Crude OR (95% CI)	*P*-value	Adjusted OR (95% CI)	*P*-value
Year				
2007	1.00		1.00	
2013	1.86 (1.51–2.29)	< 0.001*	2.21 (1.30–3.75)	0.003*
2016	4.14 (3.14–5.46)	< 0.001*	3.01 (3.83–11.67)	0.001
Age at first birth	1.01 (0.99–1.03)	0.096	Ns	
Age				
15–19	1.00		1.00	
20–24	0.86 (0.73–1.01)	0.053	0.81 (0.67–0.99)	0.045*
25–29	0.77 (0.65–0.91)	0.002*	0.79 (0.63–0.98)	0.034*
30–34	0.72 (0.61–0.84)	< 0.001*	0.85 (0.68–1.07)	0.175
35–39	0.73 (0.61–0.86)	< 0.001*	0.92 (0.70–1.21)	0.567
40–44	0.58 (0.48–0.71)	< 0.001*	0.91 (0.66–1.26)	0.577
45–49	0.50 (0.37–0.67)	< 0.001*	1.08 (0.71–1.64)	0.725
Region				
Morovia	1.00		1.00	
North Western	0.68 (0.53–0.87)	0.002*	0.76 (0.56–1.05)	0.094
South Central	0.48 (0.37–0.62)	< 0.001*	1.20 (0.87–1.66)	0.255
South Eastern A	0.76 (0.55–1.04)	0.084	1.21 (0.84–1.73)	0.303
South Eastern B	0.50 (0.39–0.65)	< 0.001*	0.94 (0.68–1.30)	0.720
North Central	0.33 (0.23–0.48)	< 0.001*	1.65 (1.04–2.61)	0.034*
Place of residence				
Urban	1.00		1.00	
Rural	0.32 (0.27–0.39)	< 0.001*	0.45 (0.31–0.65)	< 0.001*
Level of education				
No education	1.00		1.00	
Primary	1.41 (1.26–1.59)	< 0.001*	1.11 (0.96–1.27)	0.150
Secondary	3.35 (2.88–3.89)	< 0.001*	1.45 (1.20–1.76)	< 0.001*
Higher	11.32 (6.85–18.69)	< 0.001*	3.72 (1.82–7.61)	< 0.001*
Religion				
Christian	1.00		1.00	
Muslim	0.97 (0.78–1.21)	0.774	1.06 (0.78–1.44)	0.697
Traditional	0.85 (0.45–1.60)	0.614	1.54 (0.63–3.82)	0.345
Nil	0.41 (0.28–0.59)	< 0.001*	0.55 (0.35–0.85)	0.007*
Sex of household head				
Male	1.00		1.00	
Female	1.40 (1.25–1.56)	< 0.001*	1.20 (1.07–1.36)	0.003*
Frequency of reading newspaper or magazine				
Not at all	1.00		1.00	
Less than once a week	2.57 (2.00–3.31)	< 0.001*	1.01 (0.77–1.31)	0.951
At least once a week	2.93 (2.28–3.78)	< 0.001*	1.17 (0.85–1.60)	0.335
Almost every day	4.76 (2.64–8.60)	< 0.001*	0.99 (0.49–1.99)	0.979
Frequency of listening to radio				
Not at all	1.00		1.00	
Less than once a week	2.18 (1.85–2.57)	< 0.001*	1.24 (1.04–1.48)	0.016*
At least once a week	2.09 (1.78–2.46)	< 0.001*	1.12 (0.95–1.32)	0.165
Almost every day	2. (2.31–3.67)	< 0.001*	1.61 (1.24–2.10)	< 0.001*
Frequency of watching television				
Not at all	1.00		1.00	
Less than once a week	2.02 (1.67–2.43)	< 0.001*	0.96 (0.79–1.17)	0.684
At least once a week	2.22 (1.77–2.79)	< 0.001*	1.06 (0.83–1.35)	0.626
Almost every day	3.16 (2.29–4.35)	< 0.001*	1.31 (0.92–1.87)	0.133
Wealth index				
Poorest	1.00		1.00	
Poorer	1.40 (1.18–1.66)	< 0.001*	1.12 (0.81–1.53)	0.501
Middle	1.91 (1.58–2.32)	< 0.001*	1.52 (1.02–2.26)	0.039*
Richer	3.16 (2.54–3.91)	< 0.001*	2.48 (1.60–3.85)	< 0.001*
Richest	5.35 (4.09–7.00)	< 0.001*	2.72 (1.66–4.46)	< 0.001*
Children ever born				
1–4	1.00		1.00	
> 4	0.65 (0.58–0.71)	< 0.001*	0.85 (0.71–1.01)	0.080
Antenatal care visits				
< 4	1.00		1.00	
4 and above	3.62 (3.16–4.14)	< 0.001*	2.90 (2.52–3.36)	< 0.001*
Year*Residence				
2007*Urban	1.00		1.00	
2013*Rural	2.17 (1.44–3.25)	< 0.001*	1.64 (1.01–2.67)	0.047*
2016*Rural	2.95 (1.74–4.99)	< 0.001*		
Year*wealth index				
2007*Poorest	1.00		1.00	
2013*Poorer	0.98 (0.68–1.41)	0.914	1.09 (0.75–1.59)	0.643
2013*Middle	0.77 (0.49–1.23)	0.276	0.94 (0.59–1.49)	0.784
2013*Richer	0.54 (0.32–0.90)	0.018*	0.82 (0.47–1.43)	0.481
2013*Richest	0.58 (0.29–1.16)	0.121	1.18 (0.56–2.49)	0.658
2016*Poorer	1.48 (0.85–2.55)	0.164		
2016*Middle	0.77 (0.41–1.43)	0.401		
2016*Richer	0.37 (0.19–0.71)	0.003*		
2016*Richest	0.27 (0.13–0.56)	< 0.001*		

*significant at *p* < 0.05

Mckelvey and Zavoina’s R2 = 0.992

### Determinants of antenatal care visits

The results showed increase in antenatal care visits over the period of study. For geographical division, women from South Eastern A had 39% significant reduction in adequate ANC when compared to Morovia. There was 49% significant reduction in ANC visits among rural women, compared to women from urban residence after adjusting for other covariates (OR = 0.51; 95% CI = 0.36–0.72).

Women with formal education had significant higher odds of adequate antenatal visits compared to those with no formal education. In addition, religious belief was significantly associated with antenatal care visits; women of Islamic belief were 1.90 times as likely to have adequate ANC when compared to the Christian women (OR = 1.90; 95%CI = 1.38–2.61). Women who use of media such as listening to radio had higher odds of adequate antenatal care visits, compared to those who do not listen to radio. More so, wealth index was significantly associated with antenatal care utilization; women from richest households were 3.04 times as likely to utilize adequate antenatal care visits compared to women from poorest households (OR = 3.04; 95%CI = 1.75–5.29). Further, there was interaction between year and place of residence; respondents from year 2013 and rural residence were 1.83 times as likely to utilize adequate ANC visits compared to respondents from 2007 and urban after adjusting for other covariates. See Table 3 for details.

**Table 3:** Multivariable logistic regression model to examine factors associated with adequate antenatal visits in Liberia, 2007–2016

Variable	Crude OR (95% CI)	*P*-value	Adjusted OR (95% CI)	*P*-value
Year				
2007	1.00	–	1.00	–
2013	1.30 (1.07–1.59)	0.010*	0.86 (0.53–1.39)	0.537
2016	1.51 (1.16–1.95)	0.002*	–	–
Age at first birth	1.01 (0.99–1.03)	0.415	Ns	–
Age				
15–19	1.00		Ns	
20–24	1.06 (0.88–1.28)	0.532	Ns	
25–29	1.05 (0.87–1.27)	0.590	Ns	
30–34	0.95 (0.79–1.17)	0.676	Ns	
35–39	1.16 (0.94–1.42)	0.173	Ns	
40–44	0.85 (0.67–1.08)	0.196	Ns	
45–49	0.78 (0.55–1.12)	0.179	Ns	
Region				
Morovia	1.00		1.00	
North Western	0.73 (0.56–0.96)	0.023	0.95 (0.69–1.31)	0.765
South Central	0.54 (0.41–0.73)	< 0.001*	1.34 (0.97–1.86)	0.080
South Eastern A	0.41 (0.31–0.54)	< 0.001*	0.61 (0.43–0.86)	0.005*
South Eastern B	0.51 (0.39–0.66)	< 0.001*	1.06 (0.77–1.47)	0.717
North Central	0.41 (0.28–0.58)	< 0.001*	1.18 (0.76–1.82)	0.456
Place of residence				
Urban	1.00		1.00	
Rural	0.41 (0.35–0.49)	< 0.001*	0.51 (0.36–0.72)	< 0.001*
Level of education				
No education	1.00		1.00	
Primary	1.33 (1.17–1.50)	< 0.001*	1.28 (1.11–1.48)	0.001*
Secondary	2.49 (2.10–2.95)	< 0.001*	1.63 (1.27–2.09)	< 0.001*
Higher	7.01 (3.58–13.71)	< 0.001*	4.33 (1.50–12.55)	0.007*
Religion				
Christian	1.00		1.00	
Muslim	1.76 (1.34–2.32)	< 0.001*	1.90 (1.38–2.61)	< 0.001*
Traditional	0.68 (0.32–1.42)	0.301	1.21 (0.57–2.59)	0.617
Nil	0.57 (0.39–0.81)	0.002*	0.78 (0.52–1.16)	0.217
Sex of household head				
Male	1.00		1.00	
Female	1.24 (1.08–1.41)	0.002*	1.13 (0.98–1.30)	0.091
Frequency of reading newspaper or magazine				
Not at all	1.00		1.00	
Less than once a week	2.38 (1.72–3.30)	< 0.001*	1.04 (0.74–1.48)	0.812
At least once a week	2.30 (1.67–3.18)	< 0.001*	0.99 (0.69–1.45)	0.990
Almost every day	7.10 (1.50–33.65)	< 0.001*	1.37 (0.27–6.94)	0.700
Frequency of listening to radio				
Not at all	1.00		1.00	
Less than once a week	2.03 (1.67–2.46)	< 0.001*	1.33 (1.09–1.62)	0.005*
At least once a week	1.70 (1.44–2.02)	< 0.001*	1.09 (0.90–1.31)	0.366
Almost every day	2.92 (2.23–3.83)	< 0.001*	1.64 (1.18–2.27)	0.003*
Frequency of watching television				
Not at all	1.00		1.00	
Less than once a week	2.26 (1.79–2.85)	< 0.001*	1.32 (1.04–1.67)	0.024
At least once a week	1.95 (1.55–2.46)	< 0.001*	1.04 (0.81–1.32)	0.769
Almost every day	3.07 (1.99–4.74)	< 0.001*	1.10 (0.68–1.79)	0.696
Wealth index				
Poorest	1.00		1.00	
Poorer	1.38 (1.16–1.64)	< 0.001*	1.11 (0.81–1.53)	0.514
Middle	2.25 (1.86–2.72)	< 0.001*	1.54 (1.11–1.85)	0.010*
Richer	3.11 (2.51–3.85)	< 0.001*	2.06 (1.36–3.12)	0.001*
Richest	5.18 (3.85–6.95)	< 0.001*	3.04 (1.75–5.29)	< 0.001*
Children ever born				
1–4	1.00		1.00	
> 4	0.77 (0.69–0.87)	< 0.001*	1.02 (0.89–1.17)	0.758
Year*Residence				
2007*Urban	1.00		1.00	
2013*Rural	2.03 (1.4–2.95)	< 0.001*	1.83 (1.21–2.75)	0.004*
2016*Rural	2.39 (1.47–3.87)	< 0.001*		
Year*wealth index				
2007*Urban	1.00			
2013*Poorer	1.11 (0.76–1.63)	0.579	1.13 (0.76–1.68)	0.560
2013*Middle	0.99 (0.65–1.52)	0.969	1.19 (0.77–1.85)	0.421
2013*Richer	0.80 (0.50–1.29)	0.368	1.15 (0.67–1.98)	0.613
2013*Richest	0.47 (0.23–0.94)	0.033*	0.79 (0.36–1.72)	0.554
2016*Poorer	1.51 (0.85–2.67)	0.162	1.47 (0.85–2.55)	0.164
2016*Middle	1.16 (0.63–2.16)	0.628	0.76 (0.41–1.42)	0.401
2016*Richer	0.48 (0.26–0.89)	0.019*	0.36 (0.19–0.71)	0.003*
2016*Richest	0.40 (0.18–0.85)	0.018*	0.26 (0.12–0.56)	0.000*

*significant at *p* < 0.05

Mckelvey and Zavoina’s R2 = 0.987

## Discussion

This paper explored the changes in maternal services utilization and associated factors with ANC visits and facility-based delivery in post-war era in Liberia. The results of the study implied a level of progress in maternal health care including adequate ANC visits and facility-based delivery in Liberia between 2007 and 2016. The progress in adequate ANC visits does not match the improvement in facility-based delivery during the study period. Whereas an improvement in the utilization of maternal healthcare could be expected over time, especially in the post-war era, significant increase in adequate ANC visits was not observed during the study period. A major explanation could be the prolonged period and nature of the war which ended after 14 years of displacement, the system might not yet recover to the expected level of full functionality. In our findings, initial percentage of maternal care utilization was low, but with a steady increase in skilled care for women between 2007 and 2016. This is in line with the reports of a previous study [8, 24].

This study has provided vast comprehensive findings in the effect of war on maternal health care services utilization in Liberia, and an in-depth association in factors associated with maternal health. Furthermore, the level of maternal health care utilization found among rural respondents and other disadvantaged groups was worrisome during the study period. The trend was consistent in prenatal care and facility-based delivery, and this finding is comparable to other studies [25]. More so, the results showed that the use of media enhanced utilization of maternal health care services. The media specifically listening to radio could help to improve the level of awareness and behavior change communication especially in rural areas, thereby making women more informed about health care intervention programmes set to improve women’s attitude towards health care services during antenatal and intrapartum care [26, 27].

Several factors were identified to be associated with maternal health care services. Good educational background, urban residence and high wealth index were among the factors identified to increase maternal health care utilization. Education was found to have positive correlation with ANC visits and facility-based delivery. This means that women with good educational background had better maternal health care utilization. This implies that knowledge about the use of ANC visits and institutional delivery could be obtained through education. A previous study has reported that the increase in female literacy was associated with a decline in maternal mortality [28]. Notwithstanding, there are other moderators that could help to aggravate access to behavior change communication. In addition, economic status of women can affect the health-care seeking behaviour in resource constrained setting, such as Liberia. In this study, wealth inequity was a major factor identified in maternal health care utilization. There was a consistent finding of respondents with high wealth index having higher chance of adequate ANC visits and institutional delivery [29–32].

Another factor identified within the study period was the geographical region and place of residence, with urban women having higher odds of facility-based delivery and adequate antenatal care; these findings are consistent with previous studies [32–36]. For maternal services utilization, the study found significant interactions between study year, wealth status and place of residence. Women in a more recent study year, though living in rural settings had higher services utilization, which could be due to increase in awareness and other interventions in improving maternal services utilization. Attendance at ANC was shown to be associated with healthcare facility delivery. Adequacy in antenatal care visits increased the utilization of facility-based delivery, as women who attended four or more ANC visits were more likely to deliver at a healthcare facility, which is consistent with previous studies [37, 38]. ANC visits provide the opportunity to give health messages which could result in better understanding and compliance by the women to utilize skilled birth attendance.

## Strengths and limitations

This study utilized multiple national datasets from two different bodies, and over a period of 10 years to establish the significant pattern or trend in maternal health care in post-war era. The sample size collected from three rounds of surveys was sufficiently large and by standard procedure which increases the external validity of the findings for women aged between 15 to 49 years in Liberia. However cross-sectional study data are inadequate to establish causality. Again, the self-reported data can be subject to recall bias among the respondents [39, 40].

## Conclusion

Though there was an improvement in formal institutional delivery and recommended ANC attendance between 2007 and 2016, the proportions are still below the global coverage target. Particularly, the utilization varied across regions, rural-urban, maternal educational level, sex of household head, media access and wealth index. We, therefore, emphasize the need for strengthening and replication of the demand and supply side interventions if the ambitious global targets of reducing neonatal mortality, maternal mortality and stillbirths are to be achieved.

## Abbreviations

ANC: Antenatal care
DHS: Demographic health survey
IRB: Institutional review board
MCIS: Multiple indicator cluster survey
MMR: Maternal mortality ratio
NDS: National drug service
RMNCAH: Reproductive, maternal, newborn, child, and adolescent health
SDGs: Sustainable development goals
